# Solution NMR of Nanoparticles in Serum: Protein Competition Influences Binding Thermodynamics and Kinetics

**DOI:** 10.3389/fphys.2021.715419

**Published:** 2021-08-17

**Authors:** Joanna Xiuzhu Xu, Nicholas C. Fitzkee

**Affiliations:** Department of Chemistry, Mississippi State University, Starkville, MS, United States

**Keywords:** nanoparticle, protein, binding, interaction, NMR, kinetics, thermodynamics

## Abstract

The spontaneous formation of a protein corona on a nanoparticle surface influences the physiological success or failure of the synthetic nanoparticle as a drug carrier or imaging agent used *in vivo*. A quantitative understanding of protein-nanoparticle interactions is therefore critical for the development of nanoparticle-based therapeutics. In this perspective, we briefly discuss the challenges and limitations of current approaches used for studying protein-nanoparticle binding in a realistic biological medium. Subsequently, we demonstrate that solution nuclear magnetic resonance (NMR) spectroscopy is a powerful tool to monitor protein competitive binding in a complex serum medium *in situ*. Importantly, when many serum proteins are competing for a gold nanoparticle (AuNP) surface, solution NMR is able to detect differences in binding thermodynamics, and kinetics of a tagged protein. Combined with other experimental approaches, solution NMR is an invaluable tool to understand protein behavior in the nanoparticle corona.

## Introduction

Various types of therapeutic nanoparticles have been approved or being evaluated in ongoing clinical trials, including a gold nanoparticles (AuNPs), silver nanoparticles, hafnium oxide nanoparticles, liposomes, polymeric nanoparticles, and so on (Evans et al., 2018; Ali et al., 2019; Anselmo and Mitragotri, 2019). The success of any nanoparticle therapy is dictated by biological interactions on its surface (Bangham et al., 1958; Vroman, 1962; Treuel et al., 2014; Böhmert et al., 2020). When nanoparticles are administered to the human body, a mixture of proteins in the blood serum will adsorb onto the nanoparticle surface, forming a layer of protein termed the “corona” (Lynch and Dawson, 2008; Milani et al., 2012). These corona proteins are ultimately detected by cells or other biomolecules. This means that the biological interactions of the NP will be determined primarily by the properties of the protein corona (i.e., the types and number of adsorbed proteins), rather than the synthetic nature of the nanoparticle itself (Madathiparambil Visalakshan et al., 2020). Studies have shown that the protein corona can impact the cell uptake, circulation time, and toxicity of the nanoparticle (Chanana et al., 2011; Yang J.A. et al., 2013; Chen et al., 2017; Liu et al., 2020). In other words, the physiological response to the nanoparticle is controlled largely by the protein corona.

Understanding this physiological response is predicated on the physical chemistry of protein-nanoparticle interactions, especially in the context of complex biological fluids. Multiple techniques exist to monitor thermodynamics and kinetics of binding for purified proteins on a uniform population of nanoparticles; however, this type of reductionist approach may not always work (Lacerda et al., 2010; Lee et al., 2020). Specifically, multiple studies have found that the binding affinity of an individual protein fails to predict its composition in the final protein corona formed in a mixture (Monopoli et al., 2011; Zhang et al., 2019). Indeed, the mixture itself will have a significant influence on the protein(s) of interest (Mahmoudi et al., 2011).

In this perspective, we discuss the challenges involved in characterizing protein-nanoparticle adsorption in complex biological fluids. Using adsorption of the GB3 protein onto gold nanoparticles (AuNPs), we demonstrate that competition in a protein mixture alters the thermodynamic and kinetic parameters of protein-nanoparticle association. We also highlight that NMR is able to measure these parameters for tagged protein(s) in a complex biological fluid *in situ*, even in the presence of 1,000 of other serum proteins. This makes NMR a useful complement to other techniques in studying the physiological interactions of nanoparticles.

## The Challenges of Studying Adsorption in Mixtures

**FIGURE 1:**
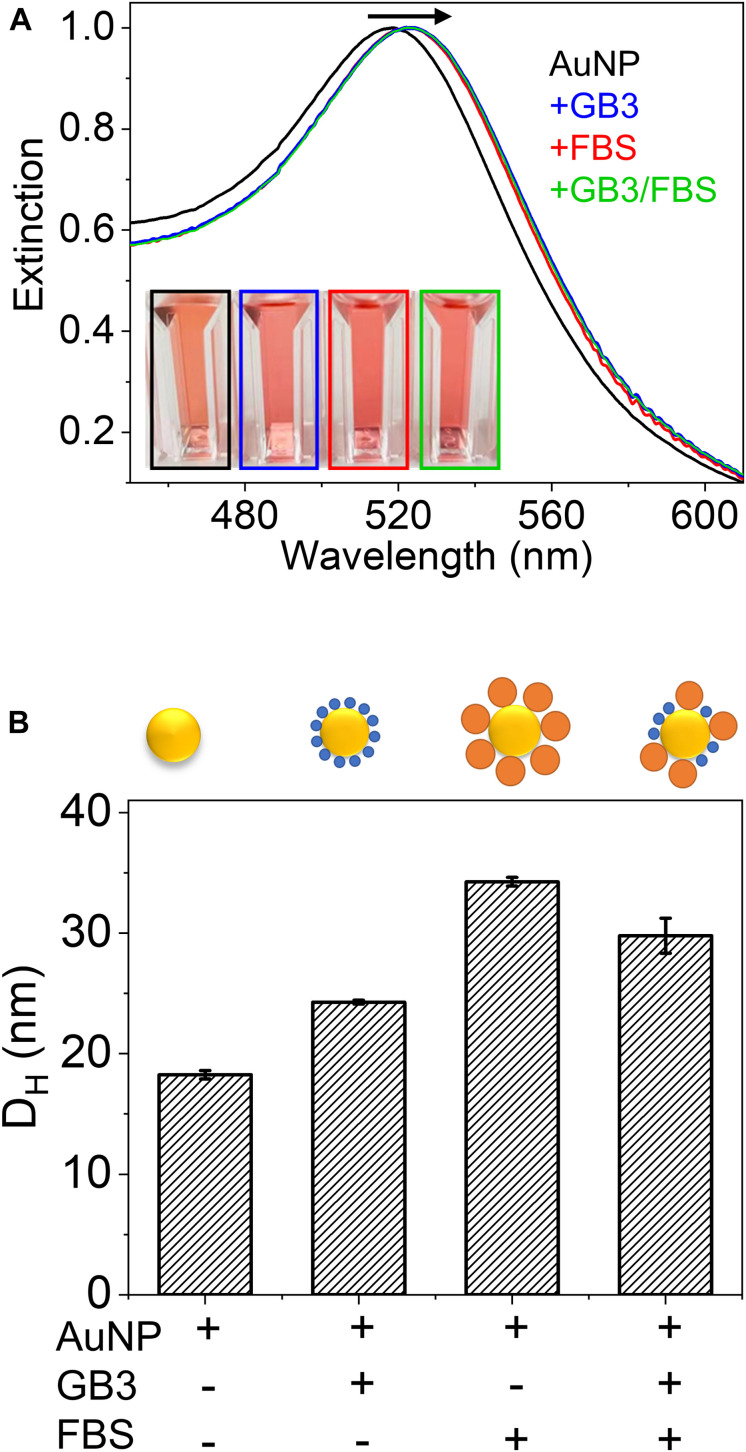
**(A)** Effect of protein coating on the UV-vis spectrum of gold nanoparticles (AuNPs). The UV-vis spectra are (black) 15-nm AuNPs (2 nM), (blue) AuNPs mixed with 0.02 mg/mL GB3, (red) AuNPs mixed with 0.02 mg/mL FBS solution, and (green) AuNPs mixed with 0.02 mg/mL GB3 and 0.02 mg/mL FBS. All spectra were normalized. **(B)** Hydrodynamic diameters (D_H_) of the four AuNP samples in **(A)** measured by DLS. The error bars represent the standard deviation of three independently prepared samples.

Optical spectroscopic techniques are commonly employed to evaluate binding of the protein of interest to a nanoparticle. For AuNPs, UV-vis spectroscopy exploits the change of the localized surface plasmon resonance (LSPR) upon surface adsorption of proteins. As illustrated in Figure 1A (experimental details in the “Supplementary Material”), when citrate-capped AuNPs are exposed to GB3, fetal bovine serum (FBS), and GB3/FBS mixtures, a red-shift of the LSPR is observed. This is because the layer of adsorbed proteins effectively changes the dielectric constant surrounding the AuNP surface (Kaur and Forrest, 2012). This phenomenon is popularly exploited to characterize the binding affinity of a protein on AuNPs, as the degree of LSPR red shift is proportional to the protein coverage on the AuNPs (Nath and Chilkoti, 2002; Yang S.- T. et al., 2013). It is worth noting that after mixing with a pure protein (GB3) or protein mixtures (FBS or GB3/FBS), the LSPR consistently shifts from 519 to 524 nm regardless of the protein identity in the corona (Figure 1A). This indicates the insensitivity of UV-vis in differentiating signals from different proteins; therefore, its applicability in studying binding of protein mixtures is severely limited.

Dynamic light scattering (DLS) is a non-invasive technique that relates the mean particle size to the timescale of fluctuations in light scattered from the nanoparticles. In protein-binding experiments, the formation of a protein corona will lead to an increase in the apparent hydrodynamic diameter (*D_H*) of the nanoparticles (Piella et al., 2017; Lee et al., 2020; McClain et al., 2020). When GB3 or FBS proteins saturate the AuNP surface, the *D_H* observed by DLS increases from 18.3 ± 0.4 nm (bare AuNP) to 24.3 ± 0.1 and 34.3 ± 0.4 nm, respectively (Figure 1B). These results reflect the fact that DLS is highly sensitive to the change in particle size resulting from protein adsorption. SDS-PAGE analysis of serum-AuNP adsorption by others demonstrates that ∼90% of adsorbed proteins are bovine serum albumin (BSA, Figure S1, Supplementary Material) (Zhang et al., 2019). It is therefore hypothesized that the majority of proteins in the FBS-AuNP corona are BSA proteins, which have a protein size ∼10 times of GB3 (6.2 kDa). Indeed, DLS detects a substantially larger *D_H* for AuNP@FBS than AuNP@GB3 (here, the @ symbol designates a core nanoparticle coated by an additional material, in this case GB3 or FBS). Interestingly, when AuNPs are exposed to a mixture of equal amount of GB3 and FBS, the resultant protein corona has a *D_H* of 29 ± 3 nm, larger than that of AuNP@GB3 but smaller than that of AuNP@FBS. This strongly suggests that competitive adsorption of GB3 and FBS proteins occurred, and both end up in the protein corona. However, further quantification of protein composition is difficult, since DLS cannot differentiate which molecules are contributing to the increase in *D_H* (James and Driskell, 2013).

Circular dichroism (CD) can be used to monitor the conformational change of a protein upon adsorption on the nanoparticle surface *in situ*. However, the background signal from the large amount of free protein makes quantitative analysis difficult, or even impossible (Yang J.A. et al., 2013). Ideally, the nanoparticle-bound protein signal should be maximized relative to the protein remaining in solution (Slocik et al., 2011); otherwise, structural analysis with CD requires knowing exact concentrations of pure species (Johnson, 1988; Micsonai et al., 2015, 2018). However, the concentrations of bound and free proteins are often unknown in samples containing nanoparticles, and as an optical technique, it is once again not straightforward to identify signals originating from individual proteins in a mixture. While CD and other optical techniques are therefore very powerful because of their ability to monitor nanoparticles *in situ*, studying individual protein behavior in complex media remains a challenge.

Non-optical approaches can also be used to study adsorption in mixtures. These approaches are often able to differentiate many proteins, but they are often unable to study nanoparticle binding *in situ*. Specifically, mass spectrometric and electrophoretic techniques are widely used to quantify multiple proteins attached in the protein corona (Monopoli et al., 2011; Mo et al., 2018; Zhang et al., 2019; Han et al., 2020; Madathiparambil Visalakshan et al., 2020; Moon et al., 2020; Pinals et al., 2020; Liessi et al., 2021) even at the detail of individual protein residues (Pustulka et al., 2020). However, these methods require extensive purification of the protein-nanoparticle complexes and complete displacement of bound proteins from the nanoparticle surface for analysis. These harsh processes, including centrifugation, are likely to alter the composition of the protein corona formed *in situ*, and complicate the interpretation of the results (Chu et al., 2021). In addition, the kinetic or thermodynamic parameters are difficult to obtain with these destructive techniques, as the protein recovery rates are low (Chu et al., 2021).

In summary, the common challenge of these methods lies in deconvoluting signals from a mixture of proteins *in situ*. As a result, many studies focus on single protein binding experiments, where competition from other proteins in the realistic biological environment are missing. Alternatively, complex mixtures are used, but the preparative approaches may disrupt the nanoparticle corona present *in situ*. To bridge this gap, a technique that is able to quantify multiprotein-nanoparticle binding *in situ* is critical for understanding nanoparticles in their physiological context.

## Solution NMR for Quantifying Protein Binding in Competition

Multi-dimensional NMR spectroscopy is an ideal non-invasive tool for quantitatively monitoring one or several proteins interacting with nanoparticles *in situ*. Several features distinguish NMR from other techniques in quantitative studies of multiprotein interactions with nanoparticles. First, only the proteins of interest that are uniquely isotopically labeled (-^2^H, -^15^N, or -^13^C) will contribute to the observed signal, while unlabeled biomolecules, buffer components, or nanoparticles will not be detected. Consequently, there is no need to separate or purify the protein corona from the nanoparticles or the biological medium as residue-specific peaks from different proteins can be identified and resolved. Second, a wealth of information on protein structure are encoded in the NMR protein peak intensities, chemical shifts, and linewidths (Assfalg et al., 2016; Perera et al., 2019). Protein NMR signals are expected to be perturbed as proteins interact with nanoparticles, depending on the strength of interaction and the properties of nanoparticles, allowing their characterization *in situ*.

**FIGURE 2:**
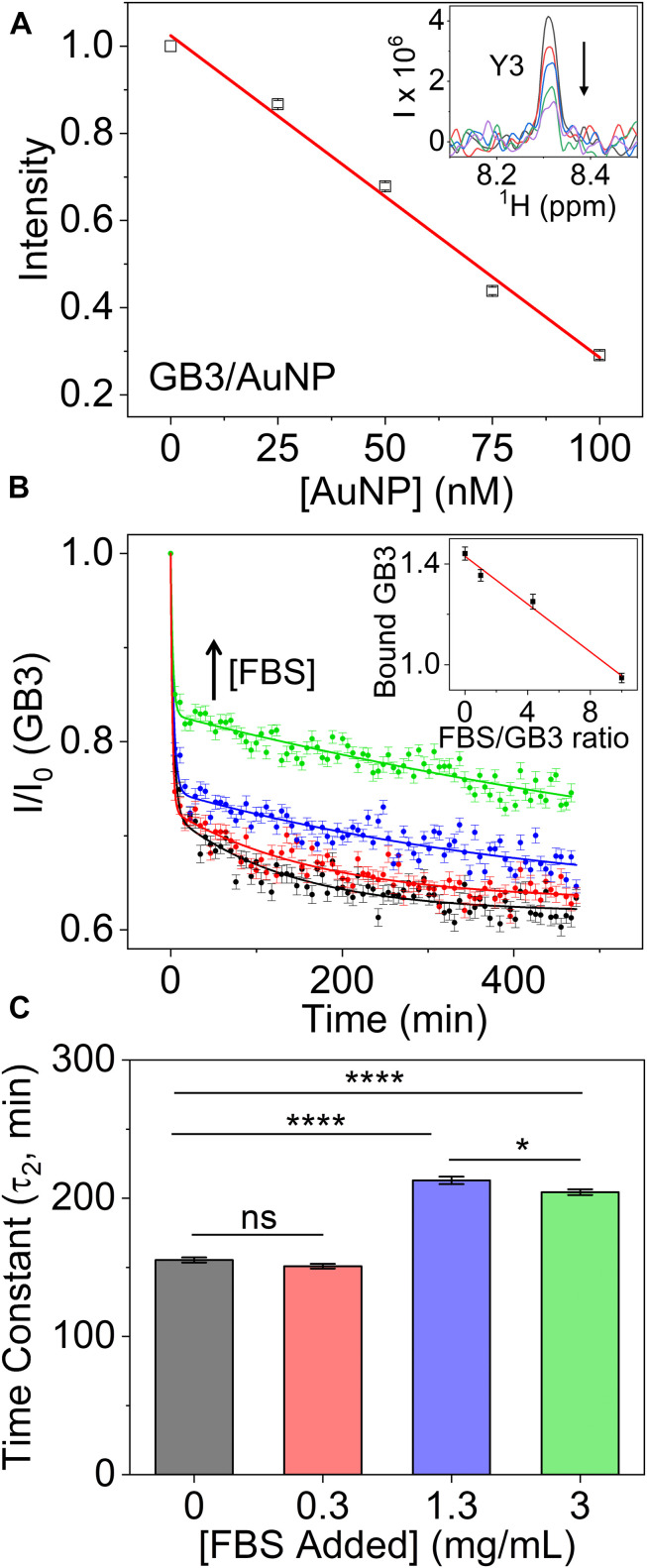
**(A)** Relative peak intensities of 20 μM GB3 as a function of increasing amount of gold nanoparticles (AuNPs) titrated into GB3 solution. The peak intensity at each titration point is averaged across 54 well- resolved peaks observed in a two-dimensional ^1^H-^15^N HSQC spectrum. The error bars represent standard deviation from three independent samples. The inset shows a cross section through one of the two dimensional peaks corresponding to residue Y3. **(B)** Effect of competitive binding on GB3 adsorption onto AuNPs in the presence of FBS. The curves show relative intensities of GB3 signals as a function of incubation time in the presence of FBS. The GB3 concentration is kept at 0.3 mg/mL, while that of FBS increases from 0 mg/mL (black), 0.3 mg/mL (red), 1.3 mg/mL (blue) to 3 mg/mL (green). A first order kinetics model with two time constants is fit to the kinetic data of GB3 adsorption (see “Supplementary Material” for details). The inset shows the bound GB3 amount (mg/mL per μM AuNPs) calculated from the relative peak intensity as a function of the FBS/GB3 concentration ratio. Each data point in the inset is the average of the last five time points, and the error bars represent the standard deviation. **(C)** Statistical comparison of the slow (τ_2_) time constants for GB3 adsorption to AuNPs in the presence of FBS. Adding FBS slows adsorption, as indicated by the longer time constants for the higher ratios of FBS to GB3. Statistical significance (*, *p* < 0.05; ****, *p* < 0.0001; ns, not significant) was determined by Tukey’s HSD test.

When protein-nanoparticle interactions are studied by solution NMR, a special case arises when the association is in the slow exchange regime. This is typically seen for the tightly bound proteins in the “hard” corona. Here, the NMR signals of nanoparticle-bound proteins will disappear entirely due to extreme line broadening, and signals from the free proteins will not exhibit line broadening at all. Under these conditions, the bound protein in the corona can be quantified using the remaining NMR signals from the free proteins (Wang et al., 2014, 2016; Perera et al., 2019). Recently, we devised a ^15^N-tryptophan (Trp) external referencing system for such a purpose in multidimensional NMR experiments (Xu et al., under review). An example of quantifying GB3 protein adsorption on 15-nm AuNPs using ^1^H-^15^N HSQC 2D NMR is depicted in Figure 2A, where the GB3 peak intensities are calibrated with Trp reference and normalized by the protein sample without AuNPs. In contrast to UV-vis or DLS, results from NMR allow quantitative calculation of the bound protein concentration. As more AuNPs are titrated into the GB3 protein solution, the GB3 peak intensities decrease linearly due to binding, and the binding capacity of GB3 can be determined to be 148 ± 8 protein per AuNP, using equations published previously (Wang et al., 2014). In addition, the peak line widths remain constant upon binding (Figure 2A, inset), which establishes the tight binding in the slow exchange regime. Conversely, the use of solid-state NMR can directly observe atomic structural details of the nanoparticle-bound proteins,(Giuntini et al., 2017) serving as a complementary technique to solution NMR.

The effect of introducing competition to protein binding is illustrated in Figure 2B, where 2D NMR is used to monitor the kinetics of binding ^15^N-labeled GB3 in the presence of FBS. The GB3 signal reduction due to adsorption at each time point was quantified by comparing signals to a control sample containing only the GB3/FBS (0 min point) (Xu et al., under review). In theory, two or more proteins can be monitored simultaneously as long as they are isotopically labeled and exhibit resolved peaks in the 2D spectrum.

Here, we show that the binding equilibrium of GB3 is significantly affected by the amount of FBS present (Figure 2B), indicating one effect of protein competition on adsorption. In particular, the bound amount of GB3 is inversely proportional to the FBS/GB3 ratio (Figure 2B, inset). The presence of other proteins in FBS reduce the final amount of GB3 adsorbed to the AuNP surface. Interestingly, however, the kinetics for GB3 adsorption are also affected. A biexponential decay was used to fit the data in Figure 2B. The baseline was constrained by averaging the final five data points for each curve, enabling a stable estimate for the fast (τ_1_) and slow (τ_2_) decay time constants (fitting details are provided in the “Supplementary Material”). The kinetic parameters are statistically different across the different conditions as assessed by an ordinary one-way ANOVA (*F*(3,196) = 232.2, *p* < 0.0001). All samples show a dramatic initial drop in intensity that occurs primarily in the experiment dead time (τ_1_), followed by a slower decay as more protein adsorbs to the nanoparticle (τ_2_). The more concentrated FBS samples have a larger τ_2_ time constant, indicating slower adsorption (Figure 2C). Given that GB3 is one of many proteins adsorbing to the AuNP surface, it is likely that its rate is slowed by the presence of other proteins proximal to the nanoparticle surface. These may be proteins in the weakly bound soft corona, which must be displaced before GB3 can bind, or they may be unbound proteins that neutralize the electrostatic environment experienced by GB3 as it approaches the AuNP surface. Clearly, the presence of FBS complicates the adsorption of GB3, and the ability to measure thermodynamic and kinetic constants *in situ* will be important in identifying the molecular interactions involved in competitive adsorption.

## Conclusion

The data presented here highlight the importance of understanding competition in protein-nanoparticle binding, and they demonstrate that quantitative NMR is an invaluable tool for understanding protein adsorption in more realistic biological environments. The mechanistic detail presented here would be challenging to obtain using other techniques, and the ability to monitor adsorption *in situ* opens new doors for understanding the molecular forces involved in protein-nanoparticle binding. While the NMR experiments presented here require the use of ^15^N isotopically labeled proteins, other options are possible, such as methylating the lysine residues of unlabeled proteins with ^13^C-formaldehyde, eliminating the need for uniform isotopic enrichment (Wang et al., 2016). Combined with traditional spectroscopy and mass-spectrometry, we believe NMR measurements such as these will be critical for understanding the nanoparticle protein corona. Given that the corona determines the physiological identity of the nanoparticle, these solution NMR approaches will lead to better nanoparticle therapeutics, and devices in the long term.

## Data Availability Statement

The raw data supporting the conclusions of this article will be made available by the authors, without undue reservation.

## Author Contributions

JX contributed to the data collection, experiment design, analysis, and manuscript preparation. NF contributed to the experiment design, analysis, and manuscript preparation. Both authors contributed to the article and approved the submitted version.

## Conflict of Interest

The authors declare that the research was conducted in the absence of any commercial or financial relationships that could be construed as a potential conflict of interest.

## Publisher’s Note

All claims expressed in this article are solely those of the authors and do not necessarily represent those of their affiliated organizations, or those of the publisher, the editors and the reviewers. Any product that may be evaluated in this article, or claim that may be made by its manufacturer, is not guaranteed or endorsed by the publisher.

## References

[B1] Ali M. R. K. Wu Y. El-Sayed M. A. (2019). Gold-nanoparticle-assisted plasmonic photothermal therapy advances toward clinical application. *J. Phys. Chem. C* 123 15375–15393. 10.1021/acs.jpcc.9b01961

[B2] Anselmo A. C. Mitragotri S. (2019). Nanoparticles in the clinic: an update. *Bioeng. Transl. Med.* 4:e10143. 10.1002/btm2.10143 PMC6764803 31572799

[B3] Assfalg M. Ragona L. Pagano K. D’onofrio M. Zanzoni S. Tomaselli S. (2016). The study of transient protein–nanoparticle interactions by solution NMR spectroscopy. *Biochim. Biophys. Acta Proteins Proteom.* 1864 102–114. 10.1016/j.bbapap.2015.04.024 25936778

[B4] Bangham A. D. Pethica B. A. Seaman G. V. F. (1958). The charged groups at the interface of some blood cells. *Biochem. J.* 69 12–19. 10.1042/bj0690012 13535575 PMC1196506

[B5] Böhmert L. Voß L. Stock V. Braeuning A. Lampen A. Sieg H. (2020). Isolation methods for particle protein corona complexes from protein-rich matrices. *Nanos. Adv.* 2 563–582. 10.1039/c9na00537d PMC9417621 36133244

[B6] Chanana M. Correa-Duarte M. A. Liz-Marzán L. M. (2011). Insulin-coated gold nanoparticles: a plasmonic device for studying metal–protein interactions. *Small* 7 2650–2660. 10.1002/smll.201100735 21913324

[B7] Chen F. Wang G. Griffin J. I. Brenneman B. Banda N. K. Holers V. M. (2017). Complement proteins bind to nanoparticle protein corona and undergo dynamic exchange in vivo. *Nat. Nanotechnol.* 12 387–393. 10.1038/nnano.2016.269 27992410 PMC5617637

[B8] Chu Y. Tang W. Zhang Z. Li C. Qian J. Wei X. (2021). Deciphering protein corona by scfv-based affinity chromatography. *Nano Lett.* 21 2124–2131. 10.1021/acs.nanolett.0c04806 33617264

[B9] Evans E. R. Bugga P. Asthana V. Drezek R. (2018). Metallic nanoparticles for cancer immunotherapy. *Mater. Today* 21 673–685. 10.1016/j.mattod.2017.11.022 30197553 PMC6124314

[B10] Giuntini S. Cerofolini L. Ravera E. Fragai M. Luchinat C. (2017). Atomic structural details of a protein grafted onto gold nanoparticles. *Sci. Rep.* 7:17934. 10.1038/s41598-017-18109-z PMC5738368 29263419

[B11] Han M. Zhu L. Mo J. Wei W. Yuan B. Zhao J. (2020). Protein corona and immune responses of borophene: a comparison of nanosheet–plasma interface with graphene and phosphorene. *ACS Appl. Bio Mater.* 3 4220–4229. 10.1021/acsabm.0c00306 35025423

[B12] James A. E. Driskell J. D. (2013). Monitoring gold nanoparticle conjugation and analysis of biomolecular binding with nanoparticle tracking analysis (NTA) and dynamic light scattering (DLS). *Analyst* 138 1212–1218. 10.1039/c2an36467k 23304695

[B13] Johnson W. C. Jr. (1988). Secondary structure of proteins through circular dichroism spectroscopy. *Annu. Rev. Biophys. Biophys. Chem.* 17 145–166. 10.1146/annurev.bb.17.060188.001045 3293583

[B14] Kaur K. Forrest J. A. (2012). Influence of particle size on the binding activity of proteins adsorbed onto gold nanoparticles. *Langmuir* 28 2736–2744. 10.1021/la203528u 22132998

[B15] Lacerda S. H. D. P. Park J. J. Meuse C. Pristinski D. Becker M. L. Karim A. (2010). Interaction of gold nanoparticles with common human blood proteins. *ACS Nano* 4 365–379. 10.1021/nn9011187 20020753

[B16] Lee J. G. Lannigan K. Shelton W. A. Meissner J. Bharti B. (2020). Adsorption of myoglobin and corona formation on silica nanoparticles. *Langmuir* 36 14157–14165. 10.1021/acs.langmuir.0c01613 33210541 PMC7735741

[B17] Liessi N. Maragliano L. Castagnola V. Bramini M. Benfenati F. Armirotti A. (2021). Isobaric labeling proteomics allows a high-throughput investigation of protein corona orientation. *Anal. Chem.* 93 784–791. 10.1021/acs.analchem.0c03134 33285070 PMC7818227

[B18] Liu N. Tang M. Ding J. (2020). The interaction between nanoparticles-protein corona complex and cells and its toxic effect on cells. *Chemosphere* 245:125624. 10.1016/j.chemosphere.2019.125624 31864050

[B19] Lynch I. Dawson K. A. (2008). Protein-nanoparticle interactions. *Nano Today* 3 40–47.

[B20] Madathiparambil Visalakshan R. González García L. E. Benzigar M. R. Ghazaryan A. Simon J. Mierczynska-Vasilev A. (2020). The influence of nanoparticle shape on protein corona formation. *Small* 16:2000285. 10.1002/smll.202000285 32406176

[B21] Mahmoudi M. Lynch I. Ejtehadi M. R. Monopoli M. P. Bombelli F. B. Laurent S. (2011). Protein-nanoparticle interactions: opportunities and challenges. *Chem. Rev.* 111 5610–5637. 10.1021/cr100440g 21688848

[B22] McClain S. M. Ojoawo A. M. Lin W. Rienstra C. M. Murphy C. J. (2020). Interaction of alpha-synuclein and its mutants with rigid lipid vesicle mimics of varying surface curvature. *ACS Nano* 14 10153–10167. 10.1021/acsnano.0c03420 32672441

[B23] Micsonai A. Wien F. Bulyáki É Kun J. Moussong É Lee Y.-H. (2018). BeStSel: a web server for accurate protein secondary structure prediction and fold recognition from the circular dichroism spectra. *Nucleic Acids Res.* 46 W315–W322. 29893907 10.1093/nar/gky497 PMC6031044

[B24] Micsonai A. Wien F. Kernya L. Lee Y.-H. Goto Y. Réfrégiers M. (2015). Accurate secondary structure prediction and fold recognition for circular dichroism spectroscopy. *Proc. Natl. Acad. Sci. U.S.A.* 112 E3095–E3103. 26038575 10.1073/pnas.1500851112 PMC4475991

[B25] Milani S. Baldelli Bombelli F. Pitek A. S. Dawson K. A. Rädler J. (2012). Reversible versus irreversible binding of transferrin to polystyrene nanoparticles: soft and hard corona. *ACS Nano* 6 2532–2541. 10.1021/nn204951s 22356488

[B26] Mo J. Xie Q. Wei W. Zhao J. (2018). Revealing the immune perturbation of black phosphorus nanomaterials to macrophages by understanding the protein corona. *Nat. Commun.* 9:2480. 10.1038/s41467-018-04873-7 PMC6018659 29946125

[B27] Monopoli M. P. Walczyk D. Campbell A. Elia G. Lynch I. Baldelli Bombelli F. (2011). Physical-chemical aspects of protein corona: relevance to in vitro and in vivo biological impacts of nanoparticles. *J. Am. Chem. Soc.* 133 2525–2534. 10.1021/ja107583h 21288025

[B28] Moon D. W. Park Y. H. Lee S. Y. Lim H. Kwak S. Kim M. S. (2020). Multiplex protein imaging with secondary ion mass spectrometry using metal oxide nanoparticle-conjugated antibodies. *ACS Appl. Mater. Interfaces* 12 18056–18064. 10.1021/acsami.9b21800 32073828

[B29] Nath N. Chilkoti A. (2002). A colorimetric gold nanoparticle sensor to interrogate biomolecular interactions in real time on a surface. *Anal. Chem.* 74 504–509. 10.1021/ac015657x 11838667

[B30] Perera Y. R. Hill R. A. Fitzkee N. C. (2019). Protein interactions with nanoparticle surfaces: highlighting solution NMR techniques. *Isr. J. Chem.* 59 962–979. 10.1002/ijch.201900080 34045771 PMC8152826

[B31] Piella J. Bastús N. G. Puntes V. (2017). Size-dependent protein–nanoparticle interactions in citrate-stabilized gold nanoparticles: the emergence of the protein corona. *Bioconj. Chem.* 28 88–97. 10.1021/acs.bioconjchem.6b00575 27997136

[B32] Pinals R. L. Yang D. Rosenberg D. J. Chaudhary T. Crothers A. R. Iavarone A. T. (2020). Quantitative protein corona composition and dynamics on carbon nanotubes in biological environments. *Angew. Chem. Int. Ed.* 59 23668–23677. 10.1002/anie.202008175 32931615 PMC7736064

[B33] Pustulka S. M. Ling K. Pish S. L. Champion J. A. (2020). Protein nanoparticle charge and hydrophobicity govern protein corona and macrophage uptake. *ACS Appl. Mater. Interfaces* 12 48284–48295. 10.1021/acsami.0c12341 33054178

[B34] Slocik J. M. Govorov A. O. Naik R. R. (2011). Plasmonic circular dichroism of peptide-functionalized gold nanoparticles. *Nano Lett.* 11 701–705. 10.1021/nl1038242 21207969

[B35] Treuel L. Brandholt S. Maffre P. Wiegele S. Shang L. Nienhaus G. U. (2014). Impact of protein modification on the protein corona on nanoparticles and nanoparticle–cell interactions. *ACS Nano* 8 503–513. 10.1021/nn405019v 24377255

[B36] Vroman L. (1962). Effect of adsorbed proteins on the wettability of hydrophilic and hydrophobic solids. *Nature* 196 476–477. 10.1038/196476a0 13998030

[B37] Wang A. Perera Y. R. Davidson M. B. Fitzkee N. C. (2016). Electrostatic interactions and protein competition reveal a dynamic surface in gold nanoparticle–protein adsorption. *J. Phys. Chem. C* 120 24231–24239. 10.1021/acs.jpcc.6b08469 27822335 PMC5096844

[B38] Wang A. Vangala K. Vo T. Zhang D. Fitzkee N. C. (2014). A three-step model for protein–gold nanoparticle adsorption. *J. Phys. Chem. C* 118 8134–8142. 10.1021/jp411543y

[B39] Yang J. A. Johnson B. J. Wu S. Woods W. S. George J. M. Murphy C. J. (2013). Study of wild-type α-synuclein binding and orientation on gold nanoparticles. *Langmuir* 29 4603–4615. 10.1021/la400266u 23477540

[B40] Yang S.-T. Liu Y. Wang Y.-W. Cao A. (2013). Biosafety and bioapplication of nanomaterials by designing protein–nanoparticle interactions. *Small* 9 1635–1653. 10.1002/smll.201201492 23341247

[B41] Zhang X. Shi H. Zhang R. Zhang J. Xu F. Qiao L. (2019). The competitive dynamic binding of some blood proteins adsorbed on gold nanoparticles. *Part. Particle Syst. Characteriz.* 36:1800257. 10.1002/ppsc.201800257

