# Supplementary material for: Solution NMR of Nanoparticles in Serum: Protein Competition Influences Binding Thermodynamics and Kinetics
Source: Front Physiol. 2021 Aug 17;12:715419. doi: 10.3389/fphys.2021.715419 (PMC8415878; doi:10.3389/fphys.2021.715419)
Supplement: Supplementary file 1 [file Data_Sheet_1.PDF]

Supplementary Material for

Solution NMR of Nanoparticles in Serum: Protein Competition Influences Binding Thermodynamics and Kinetics

Joanna Xiuzhu Xu[1], Nicholas C. Fitzkee[*1]

[1] Department of Chemistry, Mississippi State University, Mississippi State, Mississippi, 39762, United States

Table of Contents

S1. Synthesis of AuNPs S2

S2. ^{15}N-GB3 Protein expression and purification S2

S3. Characterization of Fetal Bovine Serum (FBS) S3

S4. Characterization of protein binding using UV-vis spectrometry and DLS S4

S5. Determination of GB3 binding capacity using ^{1}H-^{15}N HSQC S5

S6. Kinetic study of GB3 competitive binding with the presence of FBS using ^{1}H-^{15}N SOFAST-HMQC S6

S7. References S8

S1. Synthesis of AuNPs

Gold (III) chloride trihydrate (product # 520918) and sodium citrate dihydrate (product # 567446) were purchased from Millipore Sigma. The synthesis was performed using the method by Frens and Turkevich.(Turkevich et al., 1951;Frens, 1973). Briefly, 2 mL of 10 mg mL^{-1} $HAuCl_4$ was diluted in 198 mL of 18.2 MΩ ultrapure Milli-Q water in a round bottom flask and the solution was heated to boil at power level 70 (70% of 120 V) in a heating mantle (Glas-Col). The round bottom flask was connected to a condenser that prevents water loss during heating, and stirring was set at 600 rpm. Immediately after the solution started boiling, the power level of the mantle was set to zero. Once boiling completely stopped, 5 ml of 1% (w/v) sodium citrate was added, and the power level was turned up to 48. The solution was heated at this new power level for 25 minutes and the color of the solution turned from light yellow to red. Subsequently, the flask was removed from the heating mantle and allowed to cool to room temperature with constant 600 rpm stirring. Finally, 900 µL of 0.5 M sodium citrate was added once the solution completely cooled down to room temperature. Immediately before use, the AuNPs were concentrated by centrifugation in 50 mL Falcon tubes at 8,000 *g* for 45 minutes at 4 °C. The clear supernatant was removed carefully, and the concentrated AuNP solution was collected. 1 uL of the concentrated AuNP solution was then diluted into a 1,000 uL solution for concentration analysis, which was determined by its UV-vis peak absorption at 520 nm using a molar absorptivity of 3.94×10^8 $M^{-1}cm^{-1}$ (Liu et al., 2007). The concentrated AuNP solution was stored at 4 °C.

S2. ^{15}N-GB3 Protein expression and purification

^{15}N-ammonium chloride was obtained from Cambridge Isotope Laboratories. Wild type (wt) ^{15}N-GB3 protein were expressed recombinantly in *E. coli* (BL21) cells and purified as described previously (Wang et al., 2016;Woods et al., 2016).

S3. Characterization of Fetal Bovine Serum (FBS)

Premium grade fetal bovine serum was purchased from VWR (#89510-194) and stored at -20℃ before use. Total protein concentration of FBS stock solution was quantified to be 36.4 mg/mL with a Pierce^{TM} BCA protein assay kit supplied by Thermo Scientific (Lot # WA316402). The protein composition of the FBS mixture was analyzed by SDS-PAGE. Briefly, the FBS stock solution was diluted 8 times (8X), 16 times (16X), 32 times (32X), and 64 times (64X) with Milli-Q water. 10 µM of each diluted FBS solution was mixed with 10 µM of SDS-PAGE sample buffer (Bio-Rad) containing 5% 2-mercaptoethanol, and then heated at 95 ℃ for 5 mins and loaded on the Mini-protein TGX gel (Bio-Rad). The electrophoresis was performed at 130 V for 45 mins.

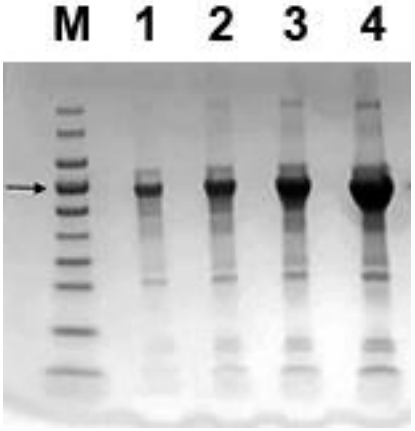

Figure S1. SDS-PAGE of a series of diluted FBS solution. The arrow highlights the 70 kDa marker, and lanes 1-4 correspond to 64X, 32X, 16X, and 8X dilutions of serum, respectively.

The most intense band located at 70 kDa on the gel (**Figure S1**) belongs to bovine serum albumin (BSA), which has a molecular weight of 66 kDa. It is therefore estimated the majority composition (>90%) of the FBS is BSA proteins.

S4. Characterization of protein binding using UV-vis spectrometry and DLS

Stock solutions of 20 nM AuNPs, 0.2 mg/mL GB3 and 0.2 mg/mL FBS were prepared. Then, three 1,000 µL protein/AuNP mixture samples were prepared by mixing 2 nM AuNPs (final concentration) with 0.02 mg/mL GB3, or 0.02 mg/mL FBS, or (0.02 mg/mL GB3 + 0.02 mg/mL FBS) mixture, respectively. The rest of solution volume was made with 10 mM HEPES at pH 7.4. A 2 nM AuNP solution in the same buffer was prepared as the control. After incubation of an hour, the UV-vis spectra of all samples were measured by an Olis-refurbished Agilent 8453 UV-Vis Spectrophotometer. Their UV peak intensities were normalized to 1 for LSPR wavelength comparison. Subsequently, the particle size of all samples was analyzed by an Anton Paar Litesizer 500 DLS at 25°C, and the data was processed using the Kalliope software.

S5. Determination of GB3 binding capacity using ^1H-^{15}N HSQC

20 µM of ^{15}N-GB3 was mixed with 0 nM, 25 nM, 50 nM, 75 nM, and 100 nM of AuNP solutions, respectively, in the presence of 10 mM HEPES at pH 7.4 and 20 mM NaCl, with a sample volume of 400 µL. After 1h incubation, the samples were transferred to a clean NMR tube (Wilmad 535-PP-7). A coaxial insert containing 2 mM ^{15}N-Tryptophan (Trp) with 7% D_2O was inserted into the NMR tube as the external intensity reference. The Trp Indole signal was used for calibrating protein peak intensities.

All NMR experiments were performed on a 600 MHz Bruker Avance III NMR system equipped with a CP-QCI cryoprobe at 25 °C. 2D ^1H-^{15}N TROSY-HSQC experiment (Pervushin et al., 1997) was used to record the 2D NMR spectra of GB3 containing different amount of AuNPs. The indirect acquisition time (t1) was 120 ms, recorded for a total of 256 complex points. The direct acquisition time (t2) was 120 ms for 1,024 points. Spectral data were processed using NMRPipe. (Delaglio et al., 1995) Both the t1 and t2 dimensions were extensively zero-filled to 4,096 and 16,384 total points respectively for quantitative analysis.

All peak intensities were normalized by the intensity of the Trp indole signal before quantitative analysis. Subsequently, the Trp-normalized peak intensity of each residue in the AuNP-containing GB3 samples (I) was normalized by that in the non-AuNP containing GB3 sample (I_0). As AuNP-bound protein peaks become invisible due to its extremely short T_2 relaxation times, (Wang et al., 2014;Perera et al., 2019) The ratio of I/I_0 corresponds to the fraction of unbound (free) protein.

S6. Kinetic study of GB3 competitive binding with the presence of FBS using ^{1}H-^{15}N SOFAST-HMQC

Four kinetic samples were prepared by mixing a series of ^{15}N-GB3/FBS mixtures with 50 nM AuNP in 10 mm HEPES at pH 7.4. The GB3/FBS mixtures were prepared by keeping GB3 concentration at a final concentration of 0.3 mg/mL and increasing FBS concentrations from 0 mg/mL, 0.3 mg/mL, 1.3 mg/mL to 3 mg/mL. Protein control samples for each kinetic sample were prepared using the same protein concentration, buffer and salt, but without the addition of AuNPs.

A SOFAST-HMQC spectrum (Schanda and Brutscher, 2005;Schanda et al., 2005;Schanda et al., 2006) was first recorded for the protein control sample after 3D shimming and tuning, and its peak intensities are referred to as intensities at 0 min (I_0). For the kinetic sample, immediately after mixing the protein mixture and AuNP solution, the sample was vortexed and transferred to an NMR tube. The coaxial Trp-reference insert was inserted, and the tube was loaded into the NMR. After 1D shimming, 80 SOFAST-HMQC spectra were collected continuously to monitor the GB3 signals (I) as a function of binding time. As each spectrum took ~ 6 mins to acquire, the total acquisition time for each kinetic sample is ~ 8 hours. The SOFAST-HMQC experiment used here employs 32 points in the indirect dimension, acquisition time of 15 ms, 16 scans, and a recycle delay of 100 ms. The processing script used for the SOFAST-HMQC experiment can be found in a previous work (Xu et al., 2021).

All residue peak intensities (both I and I_0) are normalized by the Trp standard prior to further analysis. A first order kinetics model with two time constants (**Eq. S1**) was fit to the data as follows:

$$I/I_0(t) = y_0 + A_1 e^{-t/\tau_1} + A_2 e^{-t/\tau_2} \qquad \text{(S1)}$$

where $I/I_0(t)$ is the Trp-normalized relative intensities of the residue peaks at the binding time of t. To reduce the number of fit parameters, the data was constrained so that y_0 was set to be the average of the final five intensities (after 8 hours of decay). In addition, the sum of $y_0 + A_1 + A_2$ was fixed at 1 so that the intensity decay starts at $I/I_0 = 1$ when $t = 0$. This leaves three free parameters, which were could be stably optimized for all fits: τ_1, τ_2, and A_2. The number of degrees of freedom for each fit was > 75 for all conditions tested.

Nonparametric bootstrap resampling was used to estimate the uncertainties in each of the fit parameters. 50 resampled datasets were used, and distributions of τ_2 for each FBS concentrations were compared via ordinary one-way ANOVA. Increasing the number of bootstrap replicates to 100 did not significantly change the distribution of τ_2 values. Tukey's honestly significant difference (HSD) test was used for comparing individual τ_2 time constants for each FBS concentration.

S7. References

Delaglio, F., Grzesiek, S., Vuister, G.W., Zhu, G., Pfeifer, J., and Bax, A. (1995). NMRPipe: A multidimensional spectral processing system based on UNIX pipes. *J. Biomol. NMR* 6**,** 277-293.

Frens, G. (1973). Controlled Nucleation for the Regulation of the Particle Size in Monodisperse Gold Suspensions. *Nat. Phys. Sci.* 241**,** 20-22.

Liu, X., Atwater, M., Wang, J., and Huo, Q. (2007). Extinction coefficient of gold nanoparticles with different sizes and different capping ligands. *Colloids Surf. B. Biointerfaces* 58**,** 3-7.

Perera, Y.R., Hill, R.A., and Fitzkee, N.C. (2019). Protein Interactions with Nanoparticle Surfaces: Highlighting Solution NMR Techniques. *Isr. J. Chem.* 59**,** 962-979.

Pervushin, K., Riek, R., Wider, G., and Wüthrich, K. (1997). Attenuated T_2 relaxation by mutual cancellation of dipole–dipole coupling and chemical shift anisotropy indicates an avenue to NMR structures of very large biological macromolecules in solution. *Proc. Natl. Acad. Sci. USA* 94**,** 12366-12371.

Schanda, P., and Brutscher, B. (2005). Very Fast Two-Dimensional NMR Spectroscopy for Real-Time Investigation of Dynamic Events in Proteins on the Time Scale of Seconds. *J. Am. Chem. Soc.* 127**,** 8014-8015.

Schanda, P., Kupče, Ē., and Brutscher, B. (2005). SOFAST-HMQC Experiments for Recording Two-dimensional Deteronuclear Correlation Spectra of Proteins within a Few Seconds. *J. Biomol. NMR* 33**,** 199-211.

Schanda, P., Van Melckebeke, H., and Brutscher, B. (2006). Speeding Up Three-Dimensional Protein NMR Experiments to a Few Minutes. *J. Am. Chem. Soc.* 128**,** 9042-9043.

Turkevich, J., Stevenson, P.C., and Hillier, J. (1951). A study of the nucleation and growth processes in the synthesis of colloidal gold. *Discuss. Faraday Soc.* 11**,** 55-75.

Wang, A., Perera, Y.R., Davidson, M.B., and Fitzkee, N.C. (2016). Electrostatic Interactions and Protein Competition Reveal a Dynamic Surface in Gold Nanoparticle–Protein Adsorption. *J. Phys. Chem. C* 120**,** 24231-24239.

Wang, A., Vangala, K., Vo, T., Zhang, D., and Fitzkee, N.C. (2014). A Three-Step Model for Protein–Gold Nanoparticle Adsorption. *J. Phys. Chem. C* 118**,** 8134-8142.

Woods, K.E., Perera, Y.R., Davidson, M.B., Wilks, C.A., Yadav, D.K., and Fitzkee, N.C. (2016). Understanding Protein Structure Deformation on the Surface of Gold Nanoparticles of Varying Size. *J. Phys. Chem. C* 120**,** 27944-27953.

Xu, J.X., Alom, M.S., and Fitzkee, N.C. (2021). Quantitative Measurement of Multi-Protein Nanoparticle Interactions using NMR Spectroscopy. *Anal. Chem.***,** Submitted.
